# Demographic, socioeconomic and disease knowledge factors, but not population mobility, associated with lymphatic filariasis infection in adult workers in American Samoa in 2014

**DOI:** 10.1186/s13071-020-3996-4

**Published:** 2020-03-12

**Authors:** Patricia M. Graves, Sarah Sheridan, Saipale Fuimaono, Colleen L. Lau

**Affiliations:** 1 College of Public Health, Medical and Veterinary Sciences, James Cook University, Cairns, QLD Australia; 2 Australian Institute of Tropical Health and Medicine, James Cook University, Cairns, QLD Australia; 3 Department of Global Health, Research School of Population Health, The Australian National University, Canberra, Australia; 4 Department of Public Health, American Samoa Department of Health, Pago Pago, American Samoa

**Keywords:** Lymphatic filariasis, American Samoa, Population mobility, Socioeconomic status, Disease knowledge, Sero-epidemiology, Surveillance, Mosquito

## Abstract

**Background:**

Prevalence of lymphatic filariasis (LF) antigen in American Samoa was 16.5% in 1999. Seven rounds of mass drug administration (MDA) programmes between 2000 and 2006 reduced antigen prevalence to 2.3%. The most efficient methods of surveillance after MDA are not clear, but testing specific at-risk groups such as adults may provide earlier warning of resurgence. The role of migration from LF endemic countries in maintaining transmission also needs investigation. Few studies have investigated knowledge about LF and how that relates to infection risk. This study aims to investigate associations between socio-demographics, population mobility, disease knowledge and LF infection risk.

**Methods:**

In 2014, we surveyed 670 adults aged 16–68 years (62% female) at two worksites in American Samoa. Sera were tested for LF antigen and antibodies (Bm14 and Wb123) by rapid test and/or ELISA. Multivariate logistic regression was used to assess association between seromarkers and demographic factors, household socioeconomic status (SES), residence, travel history, and knowledge of LF.

**Results:**

Overall, 1.8% of participants were positive for antigen, 11.8% for Bm14, 11.3% for Wb123 and 17.3% for at least one antibody. Recent travel outside American Samoa was not associated with positivity for any seromarker. Men had higher seroprevalence than women for all outcomes (any antibody: adjusted odds ratio (aOR) = 3.49 (95% CI: 2.21–5.49). Those aged over 35 years (compared to 15–24 years) had higher prevalence of Bm14 antibody (aOR = 3.75, 3.76 and 4.17 for ages 35–44, 45–54 and ≥ 55 years, respectively, *P* < 0.05). Lower SES was associated with seropositivity (antigen: aOR = 2.89, 95% CI: 1.09–7.69; either antibody: aOR = 1.51, 95% CI: 1.12–2.05). Those who knew that mosquitoes transmitted LF had lower Wb123 antibody prevalence (aOR = 0.55, 95% CI: 0.32–0.95).

**Conclusions:**

Opportunistic sampling of adults at worksites provided an efficient and representative way to assess prevalence and risk factors for LF in American Samoa and in hindsight, foreshadowed the resurgence of transmission. Risk of LF infection, detected by one or more serological markers, was not related to recent travel history, but was strongly associated with male gender, older age, lower SES, and lack of knowledge about mosquito transmission. These results could guide future efforts to increase MDA participation.
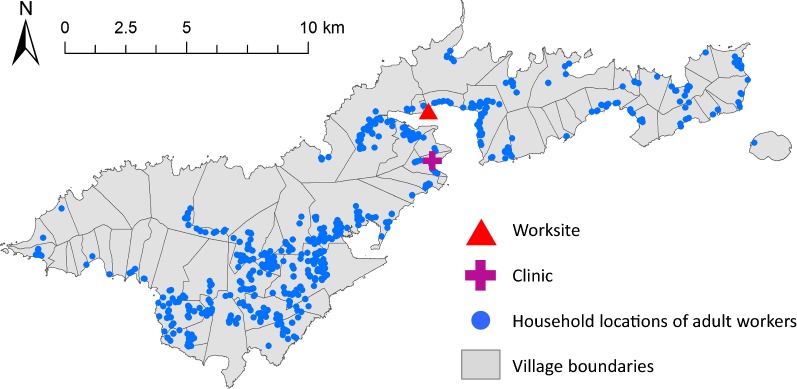

## Background

American Samoa is a US territory in the South Pacific Ocean. The population was estimated at 61,181 in 2014 [1]. There are close family and cultural ties to nearby Samoa (formerly Western Samoa) with extensive and frequent movement of people in both directions for short- and long-term visits [2], as well as to and from Hawaii and mainland USA.

American Samoa is known to have had a very high prevalence of lymphatic filariasis (LF) in the past [3]. This disease is caused by *Wuchereria bancrofti* worms which reside in the lymph system, where adult worms live for around five years and, if mated, produce microfilariae (Mf) that circulate in peripheral blood. Damage to the lymphatic system can result in lymphoedema and scrotal hydrocoele. In American Samoa, LF is classified as ‘diurnally sub-periodic’ (Mf are present in the peripheral blood at all times, but at higher density in the daytime) and is transmitted by *Aedes* mosquitoes, mainly the day-biting *Aedes polynesiensis* and also other potential day- and night-biting vectors [4, 5].

Control efforts using mass administration of diethylcarbamazine (DEC) started in American Samoa in 1962 [6], but were not consistently applied, and did not eliminate transmission of the disease. The Global Programme to Eliminate LF (GPELF) began in 2000, under auspices of the Pacific Programme for the Elimination of Lymphatic Filariasis (PacELF), offering mass drug administration (MDA) with diethylcarbamazine (DEC) and albendazole to everyone over two years of age (excluding pregnant women and the very ill) annually for at least five years [7].

Current guidelines for surveillance under the GPELF call for three sequential Transmission Assessment Surveys (TAS) in children aged 6–7 years, with at least two years between surveys [8]. The first TAS is used to determine whether MDA can be stopped, and the subsequent TAS are intended to confirm that transmission has been interrupted, if the number of positive children is below a threshold corresponding to 2% prevalence where *Anopheles* or *Culex* is the principal vector, and 1% if *Aedes* is the main vector. American Samoa’s LF programme predated the WHO 2011 guidelines [8] and the territory was following previous global and PacELF guidelines [3].

The recommended test for LF infection in *W. bancrofti* areas at the time of this study was the rapid Alere ICT (www.alere.com), which detects circulating antigen from adult worms [8]. In American Samoa, daytime blood films can also be used to detect Mf, which are present in a proportion of antigen positive individuals. Mf are cleared rapidly after effective treatment while antigen persists for many months or years; hence the focus on young children in TAS to detect recent infections. Antibody tests using Bm14 or Wb123 antibodies are also under consideration as potential surveillance markers [9], but they also persist for an uncertain period (many years) after treatment.

A seroprevalence survey in American Samoa in 2000 demonstrated an antigen prevalence of 16.5% (using Binax Now, a precursor to Alere ICT), which had declined to 2.3% in 2007 after seven rounds of MDA [10]. However, transmission was not interrupted and new infections continued to occur in both children and adults, as shown by subsequent research surveys in 2010 [11], 2014 [12] and 2016 [13], and by TAS conducted as part of programmatic activities in 2011, 2015 and 2016 [9, 13]. The persistent high prevalence in 2016, the presence of hotspots of transmission, and apparent resurgence of infection rates has led to the use of the new triple drug strategy (ivermectin, DEC and albendazole, or IDA), with the first round distributed in 2018. The triple drug strategy was recently recommended by the WHO for countries that have not achieved elimination targets despite conducting required numbers of MDA rounds [14].

LF is a heterogeneous disease with regard to gender, age and geographical distribution [11, 15–18]. Exposure to mosquitoes varies greatly depending on proximity to larval habitats, rainfall and temperature (for which altitude may be a proxy). Culturally, people in the Pacific Islands generally spend a significant amount of time outdoors and have close contact with their environment. While the vectors have a short flight range, people in American Samoa, as in other Pacific Islands, are highly mobile, both on a daily commuting basis to work at major employers [2] and to Samoa, other Pacific Islands and the USA, especially Hawaii. Other potential risk factors, such as population or household size and density, income/socioeconomic status, or work location (indoor or outdoor) [11] are not well studied. Although most people in American Samoa live in improved housing supplied with electricity, toilets and running water, there is variation in living conditions, type of sanitation, screening of windows and use of mosquito nets. Despite the longstanding presence of LF disease in American Samoa, it is not clear how much people know about the disease, or whether educational level or disease knowledge is associated with infection.

In 2014, we conducted a survey of LF infection in American Samoa in suspected hotspots, a school and worksites. Initial sero-epidemiological results of this study have been previously reported [12] in the context of confirming hotspots. Using data from the survey in worksites only, the present study aims to investigate the influence of previously uncharacterized socioeconomic status, education, work location, population mobility and knowledge of LF on the risk of LF infection.

## Methods

### Study location

American Samoa consists of 7 tropical islands and islets, with the main island Tutuila comprising 58 square miles (150 km^2^). From 2005 to 2015, annual rainfall ranged from 297 to 381 cm and average temperature was between 27.3–28.5 °C [1]. The wet tropical environment and resulting high density of mosquitoes [19] leads to high risk of vector-borne diseases; dengue seroprevalence was over 95% in 2010 [20] and there are frequent outbreaks of arboviral diseases [21, 22]. The population, estimated at 61,181 in 2014 [1] declined between 2000 and 2010 at an estimated annual rate of − 3.1%. The median age in the 2010 census was 22.4 years and the estimated per capita income was USD 6311 [1]. Major income sources are tuna canning and government services. More than 40% of the resident American Samoa population were born overseas (30% in Samoa and 6% in USA), and for over half of the population, both parents were born overseas [1]. Two-thirds of the population are either citizens or nationals of the USA who can travel there freely. Although American Samoa is geographically isolated, the population is highly mobile and travel frequently to visit family and friends, and for work, study and recreation. However, most families live on ancestral land, and permanent relocations within the territory are relatively rare [2]. American Samoa is divided administratively into 9 counties, each consisting of several villages [23].

### Study design

The study was conducted between July and November 2014 in Tutuila, American Samoa, where > 95% of the population live. Adults (*n* = 498) of working age (aged ≥ 16 years) were recruited from Starkist tuna cannery (at that time, the largest private employer in the territory) over four days in October 2014. A further 172 adults were recruited from a Department of Health (DOH) clinic where workers attend for fitness-for-work medical examinations, on 14 clinic dates (8 days between July 2 and August 15, 2014 and a further 6 days in November 2014). At both locations, convenience sampling was used, and participation was optional after informed written consent. Cannery workers attended during their breaks; DOH clinic attendees were invited to participate following routine employment checks. Interviews were conducted in Samoan or English according to participant preference, using locally appropriate terms for diseases and symptoms.

Apart from demographic characteristics and educational level achieved, participants were asked about village of residence, household location and characteristics, duration of residence in American Samoa, travel history outside of American Samoa in past 12 months, and hosting overnight visitors from Samoa in the past 12 months. They were also asked about knowledge of symptoms and causes of LF, history of taking MDA either in American Samoa or elsewhere and whether they had previously been diagnosed with LF.

### Socioeconomic status index

Most residents of American Samoa live in houses made of solid (block or wood) construction with electricity and toilets. A small proportion of households consist of traditional open buildings without walls (‘fales’). People in more conventional households may also have ‘fales’ where they spend time during the day and evening. Since participants in this survey were not interviewed at home, and their houses could not be directly observed, we developed an index measure of socioeconomic status based on data obtained through interviews. A household wealth index was constructed using the methods of Vyas & Kumaranayake [24], employing principal components analysis to group households according to similarity in their scores on nine variables: possession of assets (television, ceiling fan, air conditioning, mosquito nets (any)); plumbing (indoor toilet, indoor shower); household characteristics (screens on windows); and services (piped hot water, garbage collection). The value of the first principal component produced by the analysis for each household was used to generate the asset index, which was then divided into three quantiles.

Participants were also asked about the household combined yearly income. Due to missing data (i.e. people not knowing or choosing not to disclose their income), the income variable was not used directly; however, correlation between this income estimate and the asset index was investigated to validate use of the quantiles of asset index in the regression.

### Seroprevalence of antigen and antibody

Venous blood samples were taken from all participants, and tested by Alere ICT (www.alere.com; Brisbane, Australia) for circulating filarial antigen. If the ICT test was positive or invalid it was repeated. All persons who tested positive for antigen were treated with DEC and albendazole according to age as per WHO and DOH guidelines. Blood slides (3 lines per slide × 20 µl) were made from those who tested ICT positive. Blood samples were allowed to clot, spun at 2000×*g* for 10 min the same day and the serum decanted and frozen at − 20 °C or lower before transport to Australia.

Serological tests were performed on serum for antigen [Og4C3 ELISA (TropBio, Cellabs, Sydney, Australia)] and antibodies [Bm14 ELISA (Cellabs) and Wb123 (in-house NIH assay or InBios (InBios International Inc., Seattle, WA, USA) ELISAs] as previously described by Lau et al. [11, 12]. All ELISAs included a standard curve; we used positive cut-offs of 32 units for Og4C3 antigen, 125 units for Bm14 and 70 units for Wb123 antibody. A person was classified as antigen-positive if positive for either ICT or Og4C3 or both; as antibody-positive if positive for either Bm14 or Wb123 or both; and as ‘any positive’ if positive for any antigen or antibody.

### Data analysis

Questionnaire data were entered into Qualtrics software (www.qualtrics.com, Sydney, Australia), cleaned, merged with serological results and imported into Stata 14 (StataCorp, College Station, TX, USA) for descriptive analysis using Chi-square or Fisher’s exact tests of proportions and logistic regression of risk factors associated with antigen or antibody positivity.

Four outcomes were used as markers of positivity for LF infection: antigen; Bm14 antibody; Wb123 antibody; and ‘any antibody’. Any risk factor showing significant association with any outcome at *P* < 0.2 was tested using multivariate logistic regression and retained if *P* remained < 0.2. County of residence was included as a fixed effect to investigate the role of unmeasured geographical or other factors. Variables with *P* ≥ 0.2 were sequentially manually removed from the multivariate logistic regression models to arrive at the best-fit and most parsimonious models. Non-significant variables (*P* ≥ 0.2) were retained in the models if they improved model fit; age-group and residence time were retained in most models.

Three models were tested: MODEL A with the most complete data after assigning knowledge variables to negative if the participant had not heard of LF (*n* = 639–642 depending on outcome); MODEL B with sociodemographic and residence/travel variables only (*n* = 639–642); MODEL C with all variables, but restricted to those who reported having heard of LF (*n* = 537–538). All models were tested with and without inclusion of county as a fixed effect to account for unmeasured geographical variation.

## Results

Questionnaire data including socioeconomic and travel data were obtained for 496 of 498 cannery workers and 150 of 172 participants from the DOH clinic (total *n* = 646). There were 25 participants with missing data on gender (2/498 at the cannery and 23/172 at the DOH clinic). Job status in the last 12 months was available for only 76/172 of the DOH clinic attendees.

### Antigen, microfilaria and antibody positivity

**Fig. 1:**
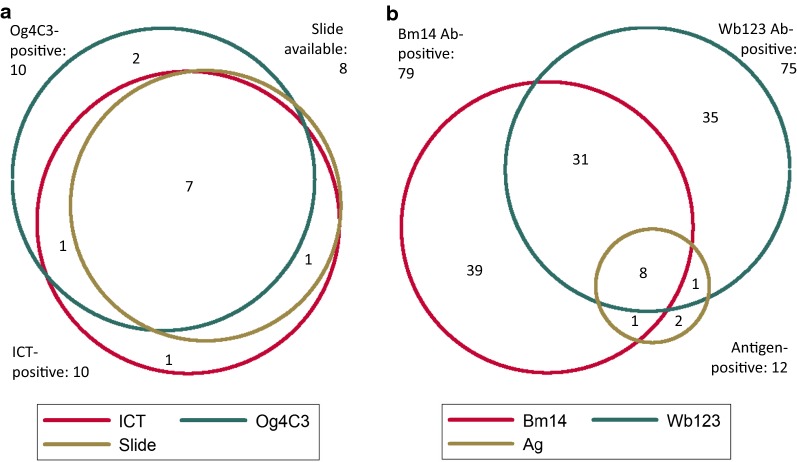
Number of positives in adult workers for **a** ICT and Og4C3 antigen, and **b** Bm14 Ab, Wb123 Ab and antigen

Overall, 1.6% (10) of 670 participants tested by ICT were positive, 1.5% (10) of 666 tested were positive by Og4C3 and 12 persons (1.8%) were positive by ICT and/or Og4C3 antigen (Fig. 1a and Table 1). Antigen prevalence was 1.6% in the cannery workers and 2.3% in the clinic attendees. Slides were available from 8 (age range 24–53 years, 4 female and 4 male) of the 10 persons who were ICT-positive (Fig. 1b); one female aged 29 years was Mf-positive with 2667 Mf/ml.

**Table 1:** Prevalence of antigen and antibody, by sampling site and overall

Site	Ag		Bm14 Ab		Wb123 Ab		Any Ab		Any positive	
	% positive (95% CI)	*n*/*N*	% positive (95% CI)	*n*/*N*	% positive (95% CI)	*n*/*N*	% positive (95% CI)	*n*/*N*	% positive (95% CI)	*n*/*N*
Cannery	1.6 (0.7–3.1)	8/498	12.5 (9.7–15.7)	62/497	12.7 (9.9–15.9)	63/497	18.3 (15.0–22.0)	91/497	18.5 (15.2–22.2)	92/497
Clinic	2.3 (0.6–5.8)	4/172	10.1 (6.0–15.6)	17/169	7.1 (3.7–12.1)	12/169	14.2 (9.3–20.4)	24/169	14.8 (9.8–21.1)	25/169
Total	1.8 (0.9–3.1)	12/670	11.9 (9.5–14.6)	75/666	11.3 (9.0–13.9)	75/666	17.3 (14.5–20.4)	115/666	17.6 (14.8–20.7)	117/666

*Abbreviations*: Ag: antigen (ICT and/or Og4C3); n, no. positive; N, no. tested

Seroprevalence of Bm14 and Wb123 antibodies were 11.9% (79/666) and 11.3% (75/666), respectively (Fig. 1b and Table 1). Overall, 17.3% of participants (115 of 666) were positive for at least one antibody, and 17.6% (117 of 666) were positive for at least one of the three seromarkers. Two people were positive for antigen but not antibody. There was no statistically significant difference in antigen, Bm14 or Wb123 antibody prevalence by sampling site (cannery or clinic).

### Demographic characteristics

#### Gender and age

**Fig. 2:**
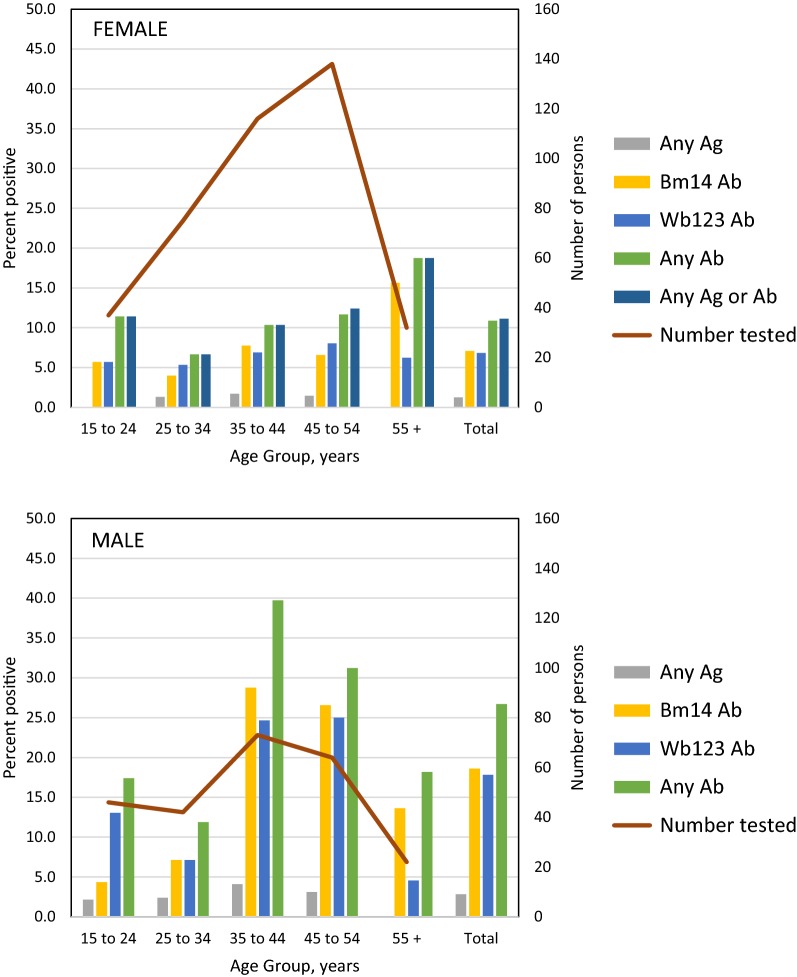
Age- and gender-specific prevalence of antigen and antibody

The prevalence of both antigen and antibody was lower in females than males in all age groups (Fig. 2). This difference was statistically significant for antibodies (Table 2). Because females were overrepresented in the sample compared to the general population (61.7% in sample *vs* 49.9% in the population [1]), the gender-adjusted prevalence of antigen was higher at 2.0% than the crude prevalence of 1.8%.

**Table 2:** Univariate analysis of demographic and socioeconomic risk factors affecting antigen and antibody positivity

Factor	Level	Ag		Bm14 Ab		Wb123 Ab		Any Ab	
		% positive (*n*)	OR (95% CI)	% positive (*n*)	OR (95% CI)	% positive (*n*)	OR (95% CI)	% positive (*n*)	OR (95% CI)
Sampling site	Cannery	1.61 (498)		12.47 (497)		12.68 (497)		18.31 (497)	
	Clinic	2.33 (172)	1.46 (0.43–4.90)	10.06 (169)	0.78 (0.44–1.38)	7.10 (169)	0.53 (0.28–1.00)**	14.20 (169)	0.74 (0.45–1.20)
Age	(per year)		1.01 (0.96–1.06)		1.03(1.00–1.05)***		1.01 (0.99–1.03)		1.01 (1.00–1.03)*
Age group (years)	15–24	1.14 (88)	Ref	6.98 (86)	Ref	11.63 (86)	Ref	16.28 (86)	Ref
	25–34	1.63 (123)	1.44 (0.13–16.11)	4.92 (122)	0.69 (0.21–2.22)	5.74 (122)	0.46 (0.17–1.27)*	8.20 (122)	0.46 (0.19–1.09)**
	35–44	2.54 (197)	2.27 (0.26–19.68)	16.75 (197)	2.68 (1.08–6.67)***	13.71 (197)	1.21 (0.56–2.62)	22.34 (197)	1.48 (0.76–2.87)
	45–54	1.94 (206)	1.72 (0.19–15.64)	12.68 (205)	1.94 (0.77–4.89)*	13.66 (205)	1.20 (0.56–2.60)	18.05 (205)	1.13 (0.58–2.22)
	> 55	0 (56)	na	14.29 (56)	2.22 (0.73–6.79)*	5.36 (56)	0.43 (0.11–1.64)	17.86 (56)	1.11 (0.46–2.73)
Gender	Female	1.26 (398)		7.09 (395)		6.84 (395)		10.89 (395)	
	Male	2.83 (247)	2.30 (0.72–7.30)*	18.62 (247)	3.00 (1.82–4.95)****	17.81 (247)	2.95 (1.78–4.91)****	26.72 (247)	2.98 (1.95–4.56)****
Ethnicity	Non-Samoan	0 (16)	#	12.50 (16)		12.50 (16)		25.0 (16)	
	Samoan	1.90 (630)	na	11.80 (627)	0.94 (0.21–4.20)	11.32 (627)	0.89 (0.20–4.01)	17.07 (627)	0.62 (0.19–1.95)
SES quantile	Per decr. quantile		2.18 (0.90–5.01)**		1.28 (0.94–1.76)*		1.43 (1.03–1.99)***		1.44 (1.09–1.89)***
SES quantile (by group)	Low	2.59 (232)	##	14.29 (231)	1.63 (0.86–3.12)	13.85 (231)	2.21 (1.08–4.52)***	21.65 (231)	2.08 (1.17–3.68)***
	Medium	2.38 (252)	na	11.20 (250)	1.24 (0.64–2.40)	11.60 (250)	1.80 (0.87–3.72)*	16.4 (250)	1.48 (0.82–2.65)*
	High	0 (162)		9.26 (162)	Ref	6.79 (162)	Ref	11.73 (162)	Ref
Persons per house	Per person		0.93 (0.75–1.15)		1.03 (0.96–1.11)		0.97 (0.89–1.05)		1.04 (0.98–1.11)
Education	Snr 2ndary or higher	2.07 (580)	#	10.92 (577)		10.75 (577)		16.46 (577)	
	< Snr 2ndary	0 (64)	na	17.19 (64)	1.69 (0.84–3.41)*	12.50 (64)	1.19 (0.54–2.60)	20.31 (64)	1.29 (0.68–2.47)
Work location	Indoor	1.69 (534)	#	11.63 (533)		11.07 (533)		16.7 (533)	
	Outdoor or mixed	0 (38)	na	18.42 (38)	1.72 (0.72–4.06)	18.42 (38)	1.81 (0.76–4.30)*	26.32 (38)	1.78 (0.84–3.80)*

# not significant (Fisher’s exact test), ## *P* < 0.04 by Fisher’s exact test comparing High *vs* Med+Low SES

*P < 0.2, **P < 0.1, ***P < 0.05, ****P < 0.01

*Abbreviations*: Ag: antigen (ICT and/or Og4C3); n, no .positive; N, no. tested; na, not available (zero cases); Snr 2ndary, senior secondary; per decr, per decreasing; Ref, reference category

Mean age for all participants was 40.0 years (95% CI: 39.1–40.8, range of 16–68 years), and 30.8% were aged 35–54 years (Fig. 2). Unadjusted estimates of antigen and antibody prevalence for all age groups are shown in Fig. 2.

The age distribution of sampled participants was previously shown to be similar to the distribution in the working population [2], but different to the general population (Additional file 1: Figure S1). Due to zero positives in some 10-year age groups, age-adjusted prevalence was estimated using two age groups: 15–34 and ≥ 35 years. Because the sampled population was older than the general adult population, the overall prevalence of antigen after adjustment for age was 1.5% compared to crude prevalence of 1.8%.

Antibody prevalence (any antibody) was lowest in age group 25–34 years in both genders. Compared to age group 15–24 years, Bm14 antibody prevalence was significantly higher in age group 35–44 years (Table 2).

#### Ethnicity

The participants were predominantly Samoan (97.5%). Other ethnicities were Filipino (1.1%), Tongan (0.9%), Tokelau (0.5%), Fijian (0.3%) and Caucasian (0.3%) (*n* = 646 with ethnicity data). There was no significant association between any outcome variable and ethnicity, but the sample sizes for non-Samoans were very small.

### Socioeconomic status characteristics

#### Socioeconomic status (SES)

Participants reported average annual household income in four categories: less than USD 10,000 (51.1% of households); USD 10,000–20,000 (35.7%); USD 20,000–30,000 (7.5%); and over USD 30,000 (5.7%). Due to a substantial proportion of people not knowing or preferring not to answer this question, information was available from only 466 (69.6%). The asset index based on 9 household items was obtained for 646 households out of a possible 670. The distribution of asset index scores and the relationship between the asset index and the reported annual income category are shown in Additional file 1: Figures S2, S3. To achieve the most complete household data, SES level was used based on asset index divided into three quantiles: low; medium; and high.

For household characteristics, 71.1% and 77.0% of households had indoor toilet and shower, respectively, 13.4% had piped hot water, and 81.3% had garbage collection. Ceiling fans were present in 92.0% of households but only 23.0% had air-conditioning; 92% of households owned a TV. The asset index included possession of two items (window screens and mosquito nets) related to vector control (although these may have limited relevance for predominantly day biting vectors). Some windows or doors were screened in 91.1% of households, while only 28.7% of households possessed at least one mosquito net. The mean number of nets per household was only 0.6 (range 0–8 nets per household) and the mean number of nets per person was 0.1.

**Fig. 3:**
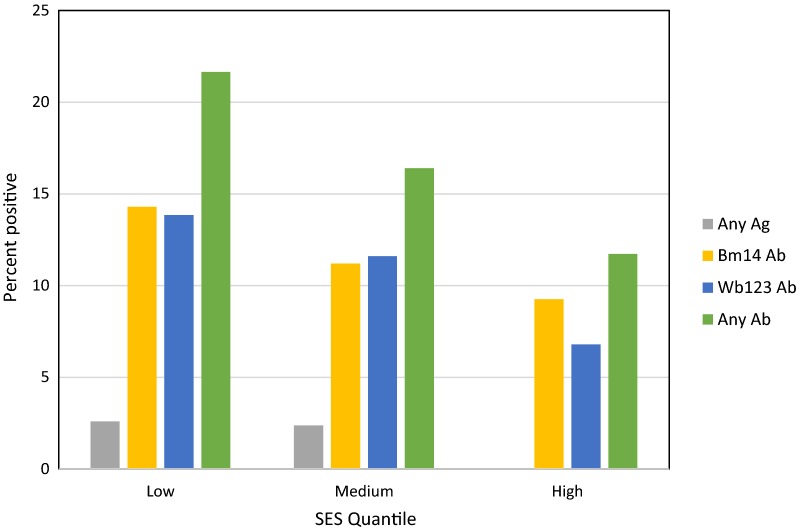
Prevalence of antigen or antibody, by SES quantile

There were no antigen-positives among persons in the highest SES quantile, compared to 2.6% and 2.4% in the lowest and medium quantiles, respectively. Antibody prevalence (both Bm14 and Wb123) was highest in the lowest SES quantile and lower in those living in households of medium and high SES (Fig. 3, Table 2).

#### Household size

Participants reported an average of 6.2 persons in their households (range 1–30); 58.8% of households were small (≤ 6 persons) and 41.2% had more than 6 persons. There was no association between household size and either antigen or antibody positivity (Table 2).

#### Educational level

In American Samoa, school attendance is mandatory for residents and comprises primary, junior secondary, and senior secondary levels, but migrants may not have attained the same educational level. There is also a post-secondary community college on Tutuila. Only 0.6% (4 persons) had no formal education, 2.8% (18) had only primary level education and 6.5% (42) only completed junior secondary. The remaining participants completed senior secondary (61.3%) or attended college/university (28.7%). Overall, 90.1% completed senior secondary school or higher-level education. Univariate analysis suggested a higher Bm14 antibody prevalence in those who had a lower level of education, but this was not statistically significant (Table 2).

#### Work status and location

Participants were asked about the location (indoors, outdoors or mixed) of their current job and of any other job they had held in the past year. Overall, 99.8% (of 574) had a job in the past year, which is not surprising since the majority were recruited at a work site (cannery) or employment clinic. Of those with known work history 36 (6.3%) and 3 (0.5%) had a second or third job in the past year, respectively, and only three people (0.5%) had a job in Samoa in the past year. The great majority of jobs (93.4%) were conducted indoors; only 1.2% and 5.4% were outdoors or mixed locations, respectively (*n* = 572). There was a tendency towards higher Wb123 antibody prevalence in those who worked outdoors (Table 2).

### Residence and mobility

#### Village and county of residence

**Fig. 4:**
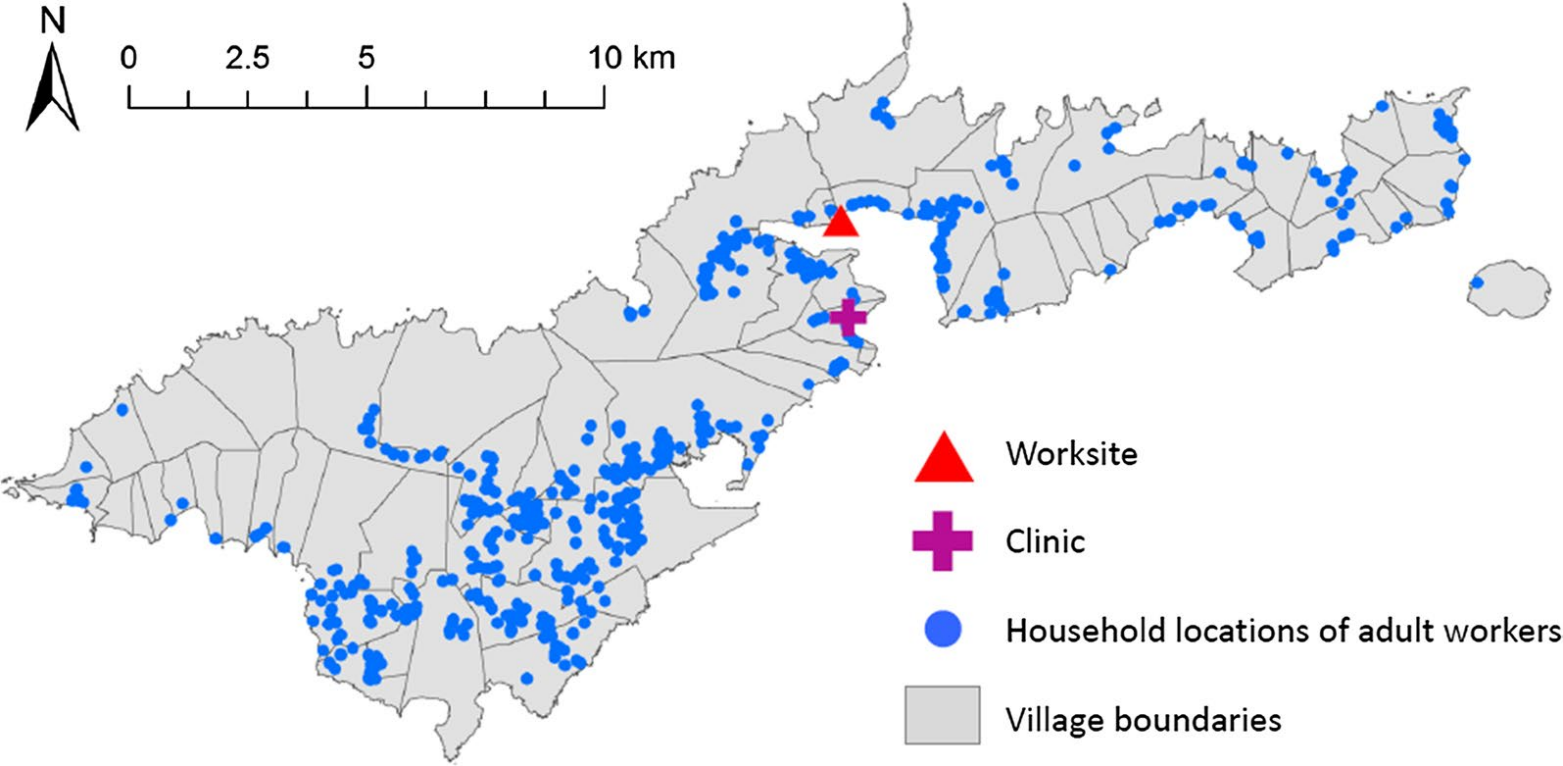
Household locations of participants recruited at worksite and clinic

Household locations of the participants were distributed throughout the islands of Tutuila and Aunu’u and are shown in relation to village boundaries in Fig. 4, which also shows the locations of the cannery and DOH clinic.

Participants lived in a total of 50 different villages (out of 74 total villages) spread across the islands of Tutuila and Aunu’u, with 1–63 participants per village. Although the cannery is located in Atu’u village, only two participants actually lived there, and most commuted from elsewhere on the two islands.

American Samoa has nine counties on Tutuila and Aunu’u; our study included 14–232 participants per county. The county of residence was assigned to each participant, to investigate whether the sample was representative of the general population. The results given in Additional file 1: Figure S4 show that the number of persons sampled was proportional to the population by county.

Prevalence of antigen and antibody varied by county (Additional file 1: Figure S5), with Tualatai county (mid-west and south of Tutuila island) having lowest prevalence of all markers while Leasina (further west) and Sua (east) counties had the highest antibody prevalence. County was not significant in univariate analysis but was included in multivariate models (see below).

#### Duration of residence in American Samoa

Only 28.9% of participants stated that they had lived in American Samoa their whole life, but the majority (70.4%) had lived there for more than 14 years. Smaller but similar proportions had lived in American Samoa for 9–14 years (13.7%) and less than 9 years (15.9%).

**Fig. 5:**
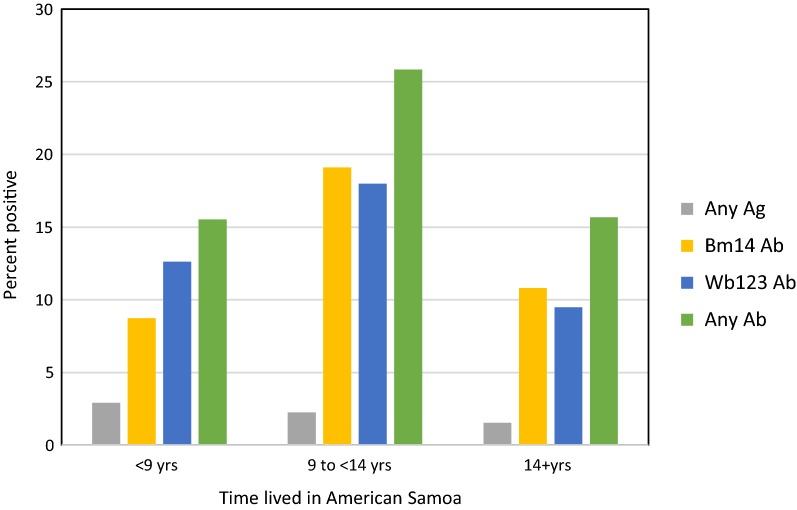
Antigen and antibody positivity, by residence time in American Samoa

Antibody prevalence was highest in persons who had lived in American Samoa for 9–14 years (Fig. 5, Table 3). While these were not necessarily continuous years before the survey, they most likely represent the time period from 2000 to 2005, i.e. during the years of MDA, which continued until 2006. Those reporting > 14 years residence were likely in American Samoa for the full duration of MDA. Those who reported residence of less than 9 years most likely missed the MDA period in American Samoa (but might have participated elsewhere) and any exposure to infection in American Samoa was probably after MDA had stopped (or occurred elsewhere).

**Table 3:** Univariate analysis of residence and mobility/travel risk factors affecting antigen and antibody positivity

Factor	Level	Ag		Bm14 Ab		Wb123 Ab		Any Ab	
		% positive (*n*)	OR (95% CI)	% positive (*n*)	OR (95% CI)	% positive (*n*)	OR (95% CI)	% positive (*n*)	OR (95% CI)
Years lived in AS	< 9	2.91 (103)	Ref	8.74 (103)	Ref	12.62 (103)	Ref	15.53 (103)	Ref
	9–14	2.25 (89)	0.77 (0.13–4.69)	19.10 (89)	2.47 (1.04–5.85)***	17.98 (89)	1.52 (0.69–3.36)	25.84 (89)	1.89 (0.93–3.87)**
	> 14	1.54 (456)	0.52 (0.13–2.04)	10.82 (453)	1.27 (0.60–2.67)	9.49 (453)	0.73 (0.37–1.41)	15.67 (453)	1.01 (0.56–1.82)
Ever been outside AS	No	2.78 (72)		6.94 (72)		9.72 (72)		11.11 (72)	
	Yes	1.72 (577)	0.62 (0.13–2.87)	12.37 (574)	1.89 (0.74–4.85)*	11.50 (574)	1.21 (0.53–2.74)	17.94 (574)	1.75 (0.81–3.76)*
Travel to Samoa^a^	No	1.86 (431)		12.12 (429)		11.66 (429)		17.72 (429)	
	Yes	1.83 (218)	0.99 (0.29–3.32)	11.06 (217)	0.90 (0.54–1.51)	10.60 (217)	0.90 (0.53–1.52)	16.13 (217)	0.89 (0.58–1.38)
Visitor from Samoa^a^	No	1.83 (383)		12.34 (381)		11.55 (381)		17.59 (381)	
	Yes	1.90 (263)	1.04 (0.33–3.32)	11.07 (262)	0.88 (0.54–1.45)	11.07 (262)	0.95 (0.58–1.57)	16.79 (262)	0.95 (0.62–1.44)
Travel to endemic area^a^	No	1.90 (421)		12.41 (419)		11.69 (419)		17.90 (419)	
	Yes	1.76 (227)	0.93 (0.28–3.11)	10.62 (226)	0.84 (0.50–1.40)	10.18 (226)	0.86 (0.51–1.45)	15.49 (226)	0.84 (0.54–1.30)
Travel to non-endemic area^a^	No	2.05 (584)	#	12.18 (583)		11.66 (583)		17.50 (583)	
	Yes	0.00 (64)	na	8.06 (62)	0.63 (0.25–1.63)	6.45 (62)	0.52 (0.18–1.48)	12.90 (62)	0.70 (0.32–1.51)

*P < 0.2, **P < 0.1, ***P < 0.05, ****P < 0.01, # not significant (Fisher’s exact test)

^a^In last 12 months

*Abbreviations*: Ag: antigen (ICT and/or Og4C3); AS, American Samoa; n, no. positive; N, no. tested; na, not available (zero cases); ref, reference category

#### Travel history

The great majority (88.9%) of the population had been outside of American Samoa (*n* = 649) at least once in their lives. Those who had ever travelled outside American Samoa had higher prevalence of Bm14 or any antibody (Table 3), but these differences were not statistically significant.

In the 12 months before our study, 38.0% of participants had travelled outside the Territory. Of these, 33.6% went to Samoa, 4.6% to other countries that were LF endemic at that time (Fiji, Tonga and Palau) and 9.9% to non-endemic overseas destinations (Hawaii, mainland USA, New Zealand, Australia, Japan). Some travellers made multiple overseas trips (mean 1.45, range 1–6 [2]), so the proportion of persons traveling to either Samoa or other endemic countries in the last year was 35.0%. In addition, 40.7% of households received visitors (who stayed at least one night) from Samoa in the last year. Most outward trips and inward visits were of one week to one month duration but ranged from less than a week to more than 6 months [2].

#### Travel to other endemic countries

The proportion of antigen-positive persons who had travelled to other endemic countries (mainly Samoa) in the last 12 months was 33.3%, in line with the overall sample. There was no increase in antigen or antibody prevalence (Bm14, Wb123 or either) in those who had travelled to other endemic countries (mainly Samoa) in the past year (Table 3, Additional file 1: Figure S6).

#### Travel to non-endemic countries

None of the people who had travelled to non-endemic countries in the last year was antigen-positive (Table 3, Additional file 1: Figure S6), compared to 2.1% in those who had not undertaken such travel. Prevalence of antibody positivity was lower in participants who had travelled to non-endemic countries in the last 12 months, but these differences were not statistically significant (Table 3).

#### Hosting visitors from Samoa

There was no difference between antigen or antibody positivity in persons living in households that had received overnight visitors from Samoa in the past year (Additional file 1: Figure S6, Table 3).

### Participation in MDA and knowledge of lymphatic filariasis

#### MDA participation

MDA has been offered repeatedly in both Samoa and American Samoa in the last 20 years, but not for ~ 8 years prior to this survey in American Samoa. Given the time elapsed, participants were reminded of the free MDA programmes run by the government, DOH and churches and asked ‘did you take tablets through this programme in American Samoa?’ or similarly ‘… in any other countries?’. Participants’ responses indicated that 62.1% had previously taken MDA in American Samoa (last round was in 2006) and 21.5% had taken it elsewhere, most likely Samoa (last nationwide round in 2011). Overall, 68.8% of persons reported taking MDA in either American Samoa or elsewhere (*n* = 618). Females were more likely to have taken MDA in the past (71% of 383 females *vs* 65% of 231 males) but this was not statistically significant (*χ*^2^ = 2.147, *df* = 1, *P* = 0.143). There was no difference in antigen or antibody prevalence by prior MDA status (Table 4 and Additional file 1: Figure S7).

**Table 4:** Univariate analysis of MDA participation, diagnosis and knowledge risk factors affecting antigen and antibody positivity

Factor	Level	Ag		Bm14 Ab		Wb123 Ab		Any Ab	
		% positive (*n*)	OR (95% CI)	% positive (*n*)	OR (95% CI)	% positive (*n*)	OR (95% CI)	% positive (*n*)	OR (95% CI)
MDA	Never taken	2.60 (192)		9.90 (192)		11.98 (192)		15.63 (192)	
	Taken (anywhere)	1.41 (426)	0.53 (0.16–1.77)	12.50 (424)	1.30 (0.75–2.26)	10.61 (424)	0.87 (0.51–1.49)	17.45 (424)	1.15 (0.72–1.81)
Ever diagnosed with LF	No	1.46 (616)	#	11.89 (614)	#	11.07 (614)		17.10 (614)	
	Yes	0.00 (8)	na	0.00 (8)	na	25.00 (8)	2.68 (0.53–13.52)	25.00 (8)	1.62 (0.32–8.12)
Know swelling or genitourinary symptom	No	2.42 (289)		12.46 (289)		12.80 (289)		19.38 (289)	
	Yes	0.78 (255)	0.32 (0.07–1.55)*	10.63 (254)	0.84 (0.49–1.42)	9.84 (254)	0.74 (0.43–1.27)	15.35 (254)	0.75 (0.48–1.18)
Know skin changes	No	1.84 (434)		12.24 (433)		11.32 (433)		18.24 (433)	
	Yes	0.91 (110)	0.49 (0.06–3.95)	9.09 (110)	0.72 (0.35–1.46)	11.82 (110)	1.05 (0.55–2.01)	14.55 (110)	0.76 (0.48–1.18)
Know chronic symptoms	No	2.49 (241)		13.69 (241)		13.28 (241)		21.16 (241)	
	Yes	0.99 (303)	0.39 (0.10–1.58)*	9.93 (302)	0.70 (0.41–1.18)*	9.93 (302)	0.72 (0.42–1.22)	14.57 (302)	0.64 (0.41–0.99)***
Know acute symptoms	No	1.11 (451)		11.09 (451)		11.31 (451)		16.85 (451)	
	Yes	4.30 (93)	4.01 (1.06–15.22)***	14.13 (92)	1.32 (0.68–2.54)	11.96 (92)	1.07 (0.53–2.13)	20.65 (92)	1.28 (0.73–2.25)
Know any symptom	No	1.52 (198)		12.63 (198)		13.64 (198)		20.71 (198)	
	Yes	1.73 (346)	1.15 (0.28–4.64)	11.01 (345)	0.86 (0.50–1.47)	10.14 (345)	0.72 (0.42–1.22)	15.65 (345)	0.71 (0.45–1.11)*
Know mosquito as cause	No	1.69 (177)		15.25 (177)		16.38 (177)		24.29 (177)	
	Yes	1.63 (367)	0.96 (0.24–3.90)	9.84 (366)	0.61 (0.35–1.03)**	9.02 (366)	0.51 (0.30–0.86)***	14.21 (366)	0.52 (0.33–0.81)***

*P < 0.2, **P < 0.1; ***P < 0.05; ****P < 0.01; # not significant (Fisher’s exact test)

*Abbreviations*: Ag: antigen (ICT and/or Og4C3); n, no. positive; N, no. tested; na, not available (zero cases)

#### Heard of LF

Overall, 84.0% (544) of the sampled population had heard of LF, out of the 648 who responded to this question. Participants age 25 and over were more likely to have heard of LF than younger participants (86.5% of 564 persons ≥ 25 years *vs* 66.7% of 84 under 25 years; *χ*^2^ = 21.398, *df* = 1, *P* < 0.001).

#### Diagnosis of LF

In this sample, eight people (1.5% of those who had heard of LF) aged between 31–54 years reported that a health worker had previously diagnosed them with LF, with year of diagnosis between 1989 and 2012 for five persons and unspecified for three persons. All eight were negative for antigen and Bm14, but two were positive for Wb123 antibody.

#### Symptoms of LF

Among those who had heard of LF before our study, over a third (37.3%) were unsure of any symptoms of LF. The unprompted response most frequently given was ‘swollen limb’ (46.9%). Very few people mentioned hydrocoele or swollen scrotum (1.7%). Summarised responses are shown in Additional file 1: Table S1. Participants aged 25 years or over were more knowledgeable about ‘any symptom of LF’ (55.3% of 564 ≥ 25 year-olds were able to correctly name a symptom of LF *vs* 40.1% of 84 under 25 year-olds; *χ*^2^ = 6.473, *df* = 1, *P* = 0.011) but differences were not significant for specific symptom categories. Females had slightly higher knowledge of all symptoms than males for most variables, but none of the differences was statistically significant.

**Fig. 6:**
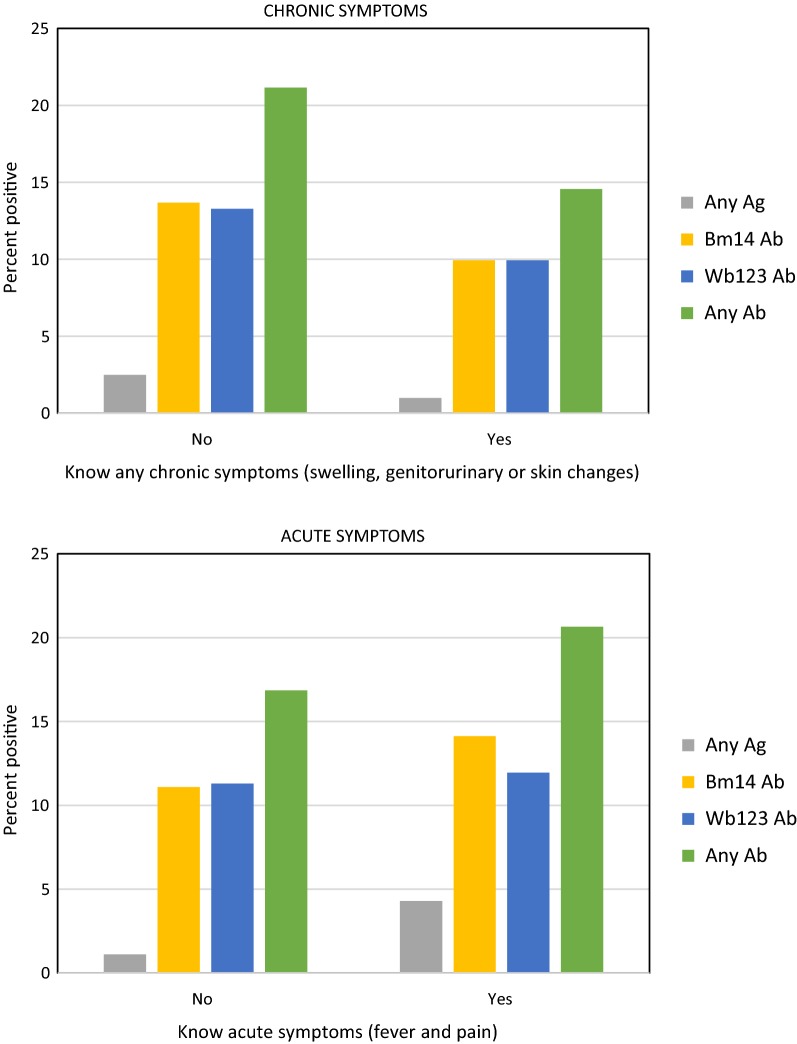
Antigen and antibody prevalence, by knowledge of LF symptoms

Antigen and antibody prevalence were lower in those who knew that chronic symptoms (swelling/genitourinary symptoms or skin changes) were related to LF (Fig. 6). The association between lower prevalence of any antibody with a combined measure of ‘knowing chronic symptoms’ was statistically significant (Table 4). Conversely, prevalence of antigen was significantly higher in those who mentioned fever and pain (classified as ‘acute symptoms’) (Fig. 6 and Table 4).

#### Causes of LF

**Fig. 7:**
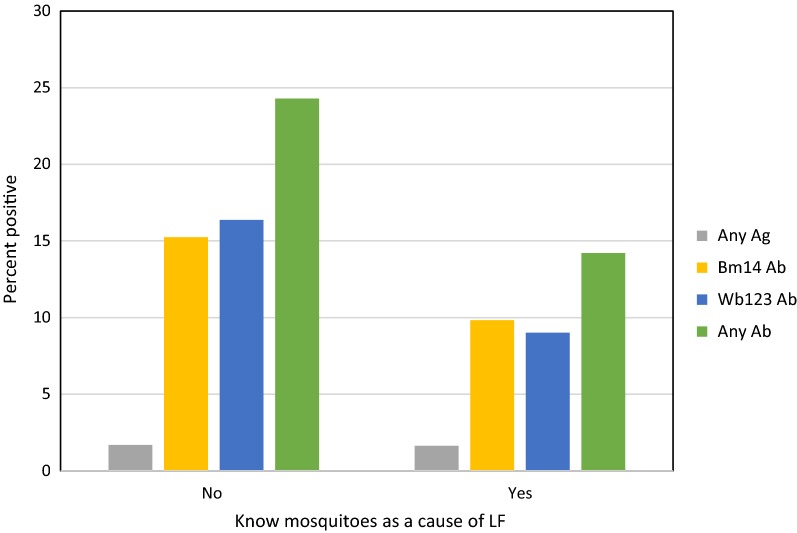
Antigen and antibody prevalence, by knowledge that mosquitoes transmit LF

Among those who had heard of LF, over a quarter (28.7%) were not able to provide any answer about the cause of the disease. Two-thirds (67.5%) of persons who had heard of LF stated the cause to be mosquito bite. Direct infection or unclean environment were mentioned by a minority of people. Summarised responses are shown in Additional file 1: Table S2. There was no significant difference between males and females in knowledge about mosquito bite as the cause of LF (65.0% of 203 males *vs* 68.6% of 338 females who had heard of LF; *χ*^2^ = 0.753, *df* = 1, *P* = 0.386). Knowledge that mosquitoes transmit LF was associated with significantly lower prevalence of Wb123 (OR = 0.51, 95% CI: 0.30–0.86, *P* = 0.013) and of any antibody (OR = 0.52, 95% CI: 0.33–0.81, *P* = 0.004) (Fig. 7 and Table 4).

### Multivariate regression analysis

Three models were tested with all four outcomes. The results of MODEL A (all participants; knowledge variables assigned to ‘no’ if person had not heard of LF) are shown in Table 5. Results for MODEL B (omitting knowledge variables to maximise sample size, Additional file 1: Table S3) and MODEL C (reduced sample size by including only those who reported knowing about LF, Additional file 1: Table S4) were similar to MODEL A (Table 5), with minor differences in adjusted odds ratios (aOR) or *P* values, or wider confidence intervals (for MODEL C). Variables were included in the final models if they had a *P* value < 0.2, or if their inclusion markedly affected the aOR of other factors or the overall model fit. We assessed the impact of including county as a fixed effect in all models, to account for unmeasured geographical or other variation and found that it decreased the log likelihood in sequential models as well as increasing the likelihood ratio and pseudo-*R*^2^ by at least 25% and up to > 100%. Therefore aORs, 95% confidence intervals and *P-*values reported in Table 5 and in Additional file 1: Tables S3 and S4 are for models including county.

**Table 5:** MODEL A: multivariate logistic regression of risk factors affecting LF antigen and antibody positivity: sociodemographic, residence and knowledge covariates, all participants (*n* = 638–641)

Factor	Level	Ag (*n* = 641)		Bm14 Ab (*n* = 641)		Wb123 Ab (*n* = 638)		Any Ab (*n* = 638)	
		OR (95% CI)	*P*	OR (95% CI)	*P*	OR (95% CI)	*P*	OR (95% CI)	*P*
Sampling site	Clinic *vs* Cannery	2.52 (0.65–9.75)*	0.182			0.52 (0.24–1.13)**	0.099		
Age group (years)	15–24			Ref		Ref		Ref	
	25–34			1.21 (0.32–4.61)	0.779	0.64 (0.21–1.94)	0.431	0.55 (0.21–1.42)	0.219
	35–44			3.75 (1.21–11.63)***	0.022	1.36 (0.54–3.40)	0.511	1.46 (0.67–3.19)	0.342
	45–54			3.76 (1.22–11.67)***	0.022	1.70 (0.69–4.21)	0.252	1.38 (0.62–3.05)	0.427
	> 55			4.17 (1.12–15.51)***	0.033	0.63 (0.15–2.70)	0.536	1.35 (0.49–3.72)	0.557
Gender	Male *vs* female	2.64 (0.78–8.94)*	0.118	3.44 (2.04–5.82)****	< 0.001	3.63 (2.11–6.27)****	< 0.001	3.49 (2.21–5.49)****	< 0.001
SES quantile	Per decr. quantile	2.89 (1.09–7.69)***	0.033	1.30 (0.92–1.84)*	0.136	1.46 (1.02–2.11)***	0.041	1.51 (1.12–2.05)****	0.007
Years lived in AS	< 9			Ref		Ref		Ref	
	9–14			2.26 (0.89–5.72)**	0.086	1.62 (0.67–3.90)	0.283	2.00 (0.91–4.40)**	0.083
	> 14			1.22 (0.55–2.70)	0.624	0.76 (0.37–1.57)	0.462	1.12 (0.58–2.13)	0.741
Ever been outside AS	Yes *vs* no							1.80 (0.75–4.32)*	0.190
Know swelling or genitourinary symptom	Yes *vs* no	0.29 (0.06–1.41)*	0.125						
Know acute symptoms	Yes *vs* no	3.53 (0.98–12.74)**	0.054					1.60 (0.86–2.95)*	0.135
Know mosquito as cause	Yes *vs* no			0.65 (0.38–1.09)*	0.102	0.55 (0.32–0.95)***	0.032	0.56 (0.36–0.90)***	0.016
Constant		0.0009 (0.0003–0.03)****	< 0.001	0.02 (0.003–0.09)****	< 0.001	0.07 (0.02–0.32)****	< 0.001	0.03 (0.01–0.13)****	< 0.001
Log-likelihood		− 52.969305		− 206.46537		− 197.11102		− 259.59574	
Log-likelihood (inc. county)		− 48.999694		− 200.41316		− 190.39068		− 253.82438	
LR Chi-square		21.25**	0.068	57.07****	< 0.001	60.62****	< 0.001	72.60****	< 0.001
Pseudo *R*^2^		0.1782		0.1246		0.1373		0.1251	

*P < 0.2, **P < 0.1, ***P < 0.05, ****P < 0.01

*Abbreviations*: Ag: antigen (ICT and/or Og4C3); AS, American Samoa; n, no. positive; N, no. tested; na, not available (zero cases); Ref, reference category; inc., including; LR, likelihood ratio

#### Antigen

The number of antigen-positives in the sample (*n* = 12) was small, limiting the power of analysis with this outcome. After adjusting for covariates, factors associated with increased antigen positivity were male gender (aOR = 2.64, 95% CI: 0.78–8.94), lower SES level (aOR = 2.89 per level, 95% CI: 1.09–7.69) and knowledge of acute symptoms (fever and/or pain) that can be due to LF (aOR = 3.53, 95% CI: 0.98–12.74) (Table 5). Sampling site (aOR = 2.52, 95% CI: 0.65–9.75 for clinic *vs* cannery site) was also retained in the model. Knowledge of some chronic LF symptoms (swelling, hydrocoele and/or chyluria) was associated with lower prevalence of antigen (aOR = 0.29, 95% CI: 0.06–1.41).

#### Antibody

For Bm14 antibody, all age groups above 35 years had higher prevalence compared to 15–24 year-olds, with aOR of 3.75, 3.76 and 4.17 (all *P* < 0.05) for ages 35–44, 45–54 and ≥ 55 years, respectively (Table 5). Other significant factors retained in the model for Bm14 prevalence were male gender (aOR = 3.44, 95% CI: 2.04–5.82), lower SES quantile (aOR = 1.30 per quantile, 95% CI: 0.92–1.84) and years of residence in American Samoa (highest in those residing for 9–14 years, compared to < 9 years, aOR = 2.26, 95% CI: 0.89–5.72). Knowing that mosquitoes transmit LF was associated with lower Bm14 antibody prevalence (aOR = 0.65, 95% CI: 0.38–1.09) (Table 5).

Wb123 antibody prevalence showed similar positive associations with male gender (aOR = 3.63, 95% CI: 2.11–6.27) and lower SES quantile (aOR = 1.46, 95% CI: 1.02–2.11) (Table 5). Other factors retained in the model that were negatively associated with Wb123 prevalence were sampling site (aOR = 0.52, 95% CI: 0.24–1.13 for clinic *vs* cannery) and knowledge that mosquitoes transmit LF (aOR = 0.55, 95% CI: 0.32–0.95).

For ‘any antibody’ (either or both Bm14 or Wb123), risk factors associated with increase in prevalence were again male gender (aOR = 3.49, 95% CI: 2.21–5.49), lower SES quantile (aOR = 1.51, 95% CI: 1.12–2.05) and years lived in American Samoa (aOR = 2.00, 95% CI: 0.91–4.40 for 9–14 years). Other factors retained were having ever travelled outside American Samoa (aOR = 1.80, 95% CI: 0.75–4.32) (although not travel or visitors in the last 12 months) and knowledge of acute symptoms (aOR = 1.60, 95% CI: 0.86–2.95). As for the individual antibody seromarkers, knowledge that mosquitoes transmit LF was associated with lower prevalence of any antibody (aOR = 0.56, 95% CI: 0.36–0.90).

## Discussion

Elimination of lymphatic filariasis as a public health problem is proving to be a challenge for GPELF in some countries. Despite MDA programmes [7] similar to those that successfully achieved validation of elimination in several PacELF countries, including Vanuatu [25], Niue [26] and Tonga [27], American Samoa has not yet achieved this milestone, even though prevalence fell to low levels by 2007 [10, 28, 29]. The processes for post-MDA surveillance and monitoring are not yet well defined, resource intensive and may fall by the wayside in the face of competing priorities. It is not clear that the current recommendations for transmission assessment surveys (TAS) in 6–7 year-old children are sensitive enough to pick up resurgent transmission until prevalence has reached high levels, and/or when transmission has become widespread. After MDA stopped in 2006, no TAS were done in American Samoa until 2011, and although TAS-1 in 2011 and TAS-2 in 2015 officially ‘passed’ the WHO targets [9], the same was not true for TAS-3 in 2016 which showed prevalence had clearly increased [13], necessitating restarting MDA with three drugs in 2018.

In the light of the current situation, we present further results from a research survey done in 2014 [12] using a different approach to routine post MDA surveillance, i.e. testing at-risk adults at work (rather than home) settings. The approach of testing adults, although not at work sites, has also been reported by Rao et al. [30] in Sri Lanka. The present study demonstrates the value of targeted surveys of working adults for efficiently providing representative information about LF prevalence and risk factors without the necessity of costly and time-consuming household cluster surveys, as have been done in many other LF endemic countries to monitor progress [31–33].

The present study confirms the highly important risk factor of gender for LF infection, with males at more than three times higher risk for antibodies. Regarding age, our sample was working-age adults only (16–68 years), with 3- to 4-fold higher prevalence of Bm14 antibody in age-groups over 35. The increased prevalence of LF in males is frequently observed worldwide, including in American Samoa [10–13] but the reasons for it are not clear. Variable exposure (e.g. increased working outside by males) alone is not likely to account for the difference [34], especially in a population engaged in a predominantly indoor occupation as in this study.

For socioeconomic status (SES), prevalence of antigen increased almost three-fold and prevalence of Wb123/any antibody increased ~ 1.5 times per SES quantile level decrease. Prior MDA (not offered in American Samoa since 2006 but available in Samoa in more recent years), educational level, and outdoor work location were not associated with presence of antigen or antibody.

There was no increased risk of LF in those who travelled to Samoa or other endemic areas in the last 12 months, or those who had household visitors from Samoa. There was also no significant decreased risk in those who had travelled to non-endemic areas in the last 12 months (mainly to Hawaii or mainland USA). The importance of these findings relate to the possibility of reintroduction or exportation of LF parasites through population mobility [2] and hence prediction of the likely success of LF elimination programmes. The prevalence in Samoa at the time of this survey was unknown, but it appears that importation of parasites from neighbouring countries (e.g. Samoa) was not a major risk factor at the time of the present study. A plausible explanation for this finding is that the two Samoas represent a single transmission zone (common pool of infection) with little difference between them in the persisting risk of infection [35]. At present, it may not be necessary to include parasite importation in spatial transmission models for American Samoa [36]. Both countries embarked in 2018 on simultaneous triple-drug MDA programmes, but if the synchronization of this slips, elimination in one Samoa will potentially be jeopardized by importation of parasites from the other in future.

A community-based study of adults in 2010 [11] observed higher prevalence in those who had lived in American Samoa for less than 5 years (compared to longer). In contrast, four years later in 2014, we did not find significantly higher seroprevalence in those who had lived a shorter time (< 9 years) in American Samoa compared to those who had lived there for 9–14 years or longer. The time periods were chosen in relation to stopping of annual MDA programmes in 2005–2006 in both Samoas. At the end of these programmes, antigen prevalence was at historically low levels [3, 10]. Those living in American Samoa for 9–14 years prior to 2014 had the highest prevalence of antibodies in the present study; they would likely have lived through some of the MDA years and received treatment, but still have persisting antibodies. Those residing in American Samoa longer than 14 years may also have received treatment from MDA during 2000–2006, but perhaps acquired infections earlier on average, and may be now losing their serological markers of infection. Further investigation of changing transmission conditions over the time period 2010–2016 and beyond is underway by modelling studies [36].

The sero-epidemiological study in 2010 [11] reported prevalence of antigen and antibodies in a sample of adults recruited at home; at that time antigen prevalence by Og4C3 ELISA was 3.2%. The antigen prevalence observed in the present study in adults (1.9% after adjusting for gender and age) was lower than in 2010, although this difference was not statistically significant (*P* = 0.08). A trend towards lower prevalence in the present study than 2010 may be due to sampling the healthy working population (with possibly lower exposure to mosquitoes) rather than the overall population in the community setting, which would include those unable to work due to LF or other disability. Even if the prevalence in working adults is an underestimate of overall adult prevalence, the results (together with the signals from the survey in 2010 [11]) could nevertheless have provided early warning of ongoing and resurgent transmission that was not identified by TAS until 2016 [13].

In the present survey, 84.0% of participants had heard of LF. This was similar to the 82.4% who had heard of LF in a community knowledge, attitudes and practice survey pre-MDA in 2003, but was a lower proportion than the 95.4% reported in an independent coverage survey after the 2004 MDA [7]. Over one-third of participants in our study could not describe any symptoms, and over a quarter were not able to provide answers about the cause of LF. It is possible that the long time elapsed since the last MDA in 2006 could have resulted in these low levels of knowledge, especially in the younger adults.

Knowledge of acute symptoms of LF was associated with higher antigen prevalence, while those who mentioned swelling or genitourinary symptoms of LF were less likely to be antigen-positive. The most common cause of LF mentioned by two-thirds of people was ‘mosquito bite’ and this knowledge was associated with lower prevalence of Bm14 and Wb123 antibodies. Knowledge of mosquito transmission of LF has previously been associated (among many other factors) with increased likelihood of taking MDA (reviewed in [37]). This knowledge may reduce LF prevalence by increasing MDA participation, but it is also possible that knowledge about the disease contributed to preventive measures against infective mosquito bites in the past.

Previously reported successful efforts to improve community participation in MDA in American Samoa included innovative communication methods to fill gaps in knowledge [7], but a long time has elapsed since 2004, and younger people would not have heard these messages or seen many people with LF morbidity. Learning from these former efforts and current findings to raise public knowledge of the symptoms and causes might lead to increased participation in the control programme and MDA in future, thus averting resurgence to pre-2000 prevalence levels.

The study has some limitations. First, a working (or work-seeking) population likely excludes those of lowest socioeconomic status and/or those who are disabled and unable to work, and is biased towards those with higher income. Nevertheless, we were able to stratify the sample by SES even in this relatively affluent population. A working population may include a higher proportion of recent migrants than the general population, although most of our participants had been in American Samoa for many years. These factors would have tended to bias the results towards lower prevalence of seromarkers and reduced likelihood of finding associations. The convenience nature of the sample was necessitated by opportunistic recruitment at the clinic and at the cannery during shift breaks, and lack of availability of full employee lists. The sample age distribution was reflective of the general working population, but was generally older and more biased towards females than the general adult population. Prior MDA participation (taking the tablets) was self-reported, and the MDA programme (conducted 8–13 years ago) did not insist on directly observed treatment at that time. We assessed travel history in the last 12 months, but seromarkers persist for many years and could have been acquired more than 12 months ago. We regard the 12-month period as a proxy marker for usual frequency of travel by participants.

Our sample was 97.5% Samoan, whereas the census 2010 [1] reported that only 85% of the population was ethnically Samoan. The prevalence of filariasis is unknown in the poorly sampled non-Samoan population who may be less likely to work in the formal sector. Efforts should be made to include them in future surveys.

Our study was novel in including socioeconomic risk factors (as is usually done in malaria surveys [38]), as well as questions about residence, mobility and knowledge of LF, and the use of both antigen and multiple antibodies for LF as outcome variables. Another strength of the study was that the sample was representative of possible residential locations, including people from all over the main islands of Tutuila and Aunu’u, encompassing the range of sociodemographic and housing conditions across the territory (at both village [2] and county levels). Sampling at worksites generated close to a simple random sample of individuals, so this type of sampling avoids the necessity of adjustment for village level clustering as needed for household cluster surveys. It was assumed that each worker lived in a different household; although it is possible that some workers (e.g. married couples) resided in the same household, this number is likely to be very small and have little influence on overall results. The workload was efficient since most of the participants were recruited over a very short time period (four days in the cannery, and added to the regular workflow on 14 specific days in the employment clinic over a 5-month period).

## Conclusions

Opportunistic sampling of adults at worksites in 2014 provided an efficient and representative way to assess prevalence and risk factors for LF in American Samoa. It confirmed the persistence of relatively high levels of infection compared to earlier surveys, and in hindsight, foreshadowed the resurgence of transmission confirmed by results from 2016. These findings suggest that current post-MDA surveillance policy that relies solely on sequential TAS in 6–7 year-olds, with the potential of failing a third TAS many years after stopping MDA, should be reviewed. The risk of being infected with LF was not related to travel history or prior MDA participation. Higher risk of infection assessed by one or more serological markers was associated with male gender, older age, lower SES, 9–14 years residence, knowledge of some chronic and acute symptoms and lack of knowledge about mosquito transmission. Future MDA should consider putting additional efforts towards achieving high drug coverage in relevant subpopulations such as adult males. To improve efficiency of surveillance and prevent resurgence after MDA has stopped, programmes should consider specifically targeting these high-risk groups, leading to higher probability of finding infected people and hotspots of continuing transmission.

## Supplementary information

**Additional file 1: Figure S1.** Distribution of the male and female population by age group (denominator is the total population aged 15 years and over, 2010 census). **Figure S2.** Frequency of households by asset index. **Figure S3.** Asset index by income category. **Figure S4.** Number of participants by county *vs* population size of county. **Figure S5.** Antigen and antibody prevalence by county. **Figure S6.** Antigen and antibody positivity, by short-term travel from and to American Samoa, last 12 months. **Figure S7.** Prevalence of antigen and antibody by whether or not MDA was taken. **Table S1.** Reported symptoms of LF. **Table S2.** Reported causes of LF. **Table S3.** MODEL B: multivariate logistic regression of risk factors affecting LF antigen and antibody positivity: sociodemographic and residence variables only (*n* = 639–642). **Table S4.** MODEL C: multivariate logistic regression of risk factors affecting LF antigen and antibody positivity: sociodemographic, residence and knowledge covariates, only participants who stated they had heard of LF (*n* = 537–540).

## Data Availability

Data supporting the conclusions of this article are provided within the article and its additional file. Raw data are available upon request from the Human Ethics Officer at the Australian National University Human Research Ethics Committee, e-mail: human.ethics.officer@anu.edu.au.

## Abbreviations

aOR: adjusted odds ratio
DEC: diethylcarbamazine
DOH: Department of Health
ELISA: enzyme-linked immunosorbent assay
GPELF: Global Programme to Eliminate Lymphatic Filariasis
ICT: immunochromatographic test
LF: lymphatic filariasis
MDA: mass drug administration
OR: odds ratio
PacELF: Pacific Programme for the Elimination of Lymphatic Filariasis
TAS: transmission assessment survey
WHO: World Health Organization
