# Supplementary material for: Demographic, socioeconomic and disease knowledge factors, but not population mobility, associated with lymphatic filariasis infection in adult workers in American Samoa in 2014
Source: Parasit Vectors. 2020 Mar 12;13:125. doi: 10.1186/s13071-020-3996-4 (PMC7068921; doi:10.1186/s13071-020-3996-4)
Supplement: Supplementary file 1 — Additional file 1: Figure S1. Distribution of the male and female population by age group (denominator is the total population aged 15 years and over, 2010 census). Figure S2. Frequency of households by asset index. Figure S3. Asset index by income category. Figure S4. Number of participants by county vs population size of county. Figure S5. Antigen and antibody prevalence by county. Figure S6. Antigen and antibody positivity, by short-term travel from and to American Samoa, last 12 months. Figure S7. Prevalence of antigen and antibody by whether or not MDA was taken. Table S1. Reported symptoms of LF. Table S2. Reported causes of LF. Table S3. MODEL B: multivariate logistic regression of risk factors affecting LF antigen and antibody positivity: sociodemographic and residence variables only (n = 639–642). Table S4. MODEL C: multivariate logistic regression of risk factors affecting LF antigen and antibody positivity: sociodemographic, residence and knowledge covariates, only participants who stated they had heard of LF (n = 537–540). [file 13071_2020_3996_MOESM1_ESM.docx]

# Additional file 1

# Graves et al. Demographic, socioeconomic and disease knowledge factors, but not population mobility, associated with lymphatic filariasis infection in adult workers in American Samoa in 2014

## Age and gender distribution in sampled population and in general population of American Samoa, 2014

**Figure S1** Distribution of the male and female population by age group (denominator is the total population aged 15 years and over, 2010 census)

## Relationship between asset index and reported household income.

**
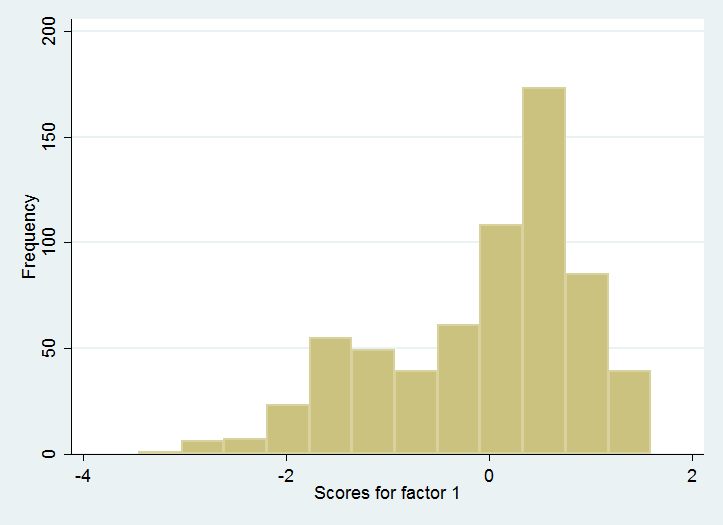
Figure S2** Frequency of households by asset index

There is a significant association between asset index and income category (t=5.47, p<0.001, Fig S3). However, there is a wide range of asset indices (reflecting past household wealth and house characteristics) in each income category. The asset index gives a more granular estimate of socioeconomic status for a larger sample. For the purposes of regression, the asset index was divided based on 3 quantiles of the index, with 34.6% of household in the lowest SES quantile 1, 37.6% in quantile 2, and 24.2% of households in the highest SES quantile 3.

**Figure S3** Asset index by income category

## Distribution of residence of sampled individuals by county

The counties with most participants were Tualauta in the central plains (includes the villages of Faleniu, Ili’Ili, Malaeimi, MapusagaFou, Pavaiai, Tafuna and Vaitogi) and Maoputasi around Pago Pago harbour (includes Atuu, Aua, Fagaalu, Fagatogo, Leloaloa, Pago Pago and Utulei villages). These are the two counties with the largest populations.

The county with fewest participants was Saole, which includes Alofau, Amouli, Auasi, Aunuu and Pagai villages in the eastern end of the main island.

**Figure S4** Number of participants by county vs population size of county

The relationship between county size and number of survey participants indicates the representative nature of the sample (Fig S4). The proportion of samples positive varied by county (Fig S5), with the lowest proportion positive in Tualatai County (includes the villages of Futiga, Taputimu and Vailoa.

**Figure S5** Antigen and antibody prevalence by county

## Short-term mobility in the year before the survey

**Figure S6** Antigen and antibody positivity, by short term travel from and to American Samoa, last 12 months.

## Participation in prior MDA

**Figure S7** Prevalence of antigen and antibody by whether or not MDA was taken

## Symptoms and causes of LF

**Table S1** Reported symptoms of LF

| **Response category** | **Number of responses*** | **% of persons** |
| --- | --- | --- |
| Swelling  Limb  Breast | 255  2 | 46.9 |
| Genitourinary  Hydrocoele  White urine | 9  1 | 1.8 |
| Skin changes  Redness  Dark skin  Other (rash, bumps, thinness, itchy) | 48  12  60 | 20.2 |
| Other symptoms  Fever  Pain  Headache  Body ache  Weakness  Other (cold symptoms, nausea, loss of appetite, coughing) | 60  51  5  2  2  4 | 18.4 |
| Don’t know or none mentioned |  | 37.3 |

*Multiple responses were allowed

**Table S2** Reported causes of LF

| **Response category** | **Number of responses*** | **% of persons** |
| --- | --- | --- |
| Mosquito bite | 367 | 67.5 |
| Infection  (catch from another person, blood, germs or pig scratch) | 18 | 3.3 |
| Environment  (unclean, hygiene, dirty water, going barefoot) | 38 | 7.0 |
| Ingestion  (food, diet, alcohol) | 9 | 1.7 |
| Other  (injury, diabetes, health care, not taking pills) | 6 | 1.1 |
| Don’t know or none mentioned | 156 | 28.7 |

*Multiple responses were allowed

## Alternative regression models

**Table S3** MODEL B: Multivariate logistic regression of risk factors affecting LF antigen and antibody positivity:
sociodemographic and residence variables only (N=639-642)

| **FACTOR** | **LEVEL** | **Ag** (N=642) | | **Bm14 Ab (**N=639) | | **Wb123 Ab (**N= 639) | | **Any Ab (**N=639) | |
| --- | --- | --- | --- | --- | --- | --- | --- | --- | --- |
|  |  | **OR (95% CI)** | **p** | **OR (95% CI)** | **p** | **OR (95% CI)** | **p** | **OR (95% CI)** | **p** |
| *Sampling* | Clinic vs |  |  |  |  | *0.56** | *0.138* |  |  |
| *site* | Cannery |  |  |  |  | *(0.26-1.21)* |  |  |  |
| **Age**  **group**  **(yrs)** | 15-24 |  |  | Ref |  | Ref |  | Ref |  |
|  | 25-34 |  |  | 1.15 | 0.833 | 0.62 | 0.397 | 0.53 | 0.184 |
|  |  |  |  | (0.0.30-4.38) |  | (0.21-1.87) |  | (0.21-1.36) |  |
|  | **35-44** |  |  | **3.46***** | **0.030** | 1.25 | 0.626 | 1.34 | 0.461 |
|  |  |  |  | **(1.12-10.67)** |  | (0.51-3.11) |  | (0.62-2.90) |  |
|  | **45-54** |  |  | **3.53***** | **0.028** | 1.59 | 0.310 | 1.32 | 0.485 |
|  |  |  |  | **(1.14-10.88)** |  | (0.65-3.91) |  | (0.61-2.88) |  |
|  | **55+** |  |  | **4.28**** | **0.030** | 0.69 | 0.616 | 1.44 | 0.474 |
|  |  |  |  | **(1.16-15.89)** |  | (0.16-2.93) |  | (0.53-3.92) |  |
| **Gender** | Male vs | *2.67** | *0.104* | **3.49****** | **<0.001** | **3.68****** | **<0.001** | **3.50****** | **<0.001** |
|  | female | *(0.82-8.76)* |  | **(2.07-5.90)** |  | **(2.14-6.34)** |  | **(2.23-5.49** |  |
| **SES** | Per | *2.26*** | *0.068* | *1.32** | *0.117* | **1.49****** | **0.032** | **1.54****** | **0.005** |
| **quantile (3)** | decreasing | *(0.94-5.43)* |  | *(0.93-1.87)* |  | **(1.03-2.15)** |  | **(1.14-2.07)** |  |
|  | quantile |  |  |  |  |  |  |  |  |
| ***Years lived in Am. Samoa*** | <9 yrs |  |  | Ref |  | Ref |  | Ref |  |
|  | 9-14 yrs |  |  | *2.30*** | *0.078* | 1.63 | 0.276 | *2.02*** | *0.078* |
|  |  |  |  | *(0.91-5.83)* |  | (0.68-3.90) |  | *(0.92-4.42)* |  |
|  | >14 yrs |  |  | 1.21 | 0.634 | 0.75 | 0.436 | 1.06 | 0.840 |
|  |  |  |  | (0.55-2.68) |  | (0.36-1.55) |  | (0.56-2.03) |  |
| *Ever been* | Yes vs |  |  |  |  |  |  | *1.81** | *0.185* |
| *outside AS* | no |  |  |  |  |  |  | *(0.75-4.33)* |  |
| **Constant** |  | **0.002****** | **<0.001** | **0.01** | **<0.001** | **0.05** | **<0.001** | **0.02** | **<0.001** |
|  |  | **(0.00009-0.04)** |  | **(0.003-0.07)** |  | **(0.01-0.22)** |  | **(0.01-0.11)** |  |
| Log likelihood |  | -56.855448 |  | -207.84677 |  | -199.60747 |  | -263.67802 |  |
| log likelihood  (inc. county) |  | -52.82362 |  | -201.75765 |  | -192.72077 |  | -257.24265 |  |
| **LR Chi^2^** |  | *13.65*** | *0.058* | **54.63****** | **<0.001** | **56.19****** | **<0.001** | **66.13****** | **<0.001** |
| Pseudo R^2^ |  | 0.1143 |  | 0.1192 |  | 0.1272 |  | 0.1139 |  |

** P<0.2; ** p<0.1;* ***** p<0.05; **** p<0.01**

**Table S4** MODEL C: Multivariate logistic regression of risk factors affecting LF antigen and antibody positivity:
sociodemographic, residence and knowledge covariates; only participants who stated they had heard of LF (N=537-540).

| **FACTOR** | **LEVEL** | **Ag** (N=538) | | **Bm14 Ab** (N=540) | | **Wb123 Ab** (N=537) | | **Any Ab** (N=537) | |
| --- | --- | --- | --- | --- | --- | --- | --- | --- | --- |
|  |  | **OR (95% CI)** | **p** | **OR (95% CI)** | **p** | **OR (95% CI)** | **p** | **OR (95% CI)** | **p** |
| Sampling | Clinic vs |  |  |  |  | 0.56 | 0.201 |  |  |
| site | cannery |  |  |  |  | (0.23-1.35) |  |  |  |
| *Age group*  *(yrs)* | 15-24 |  |  | Ref |  | Ref |  | Ref |  |
|  | 25-34 |  |  | 1.25 | 0.769 | 0.52 | 0.284 | 0.54 | 0.237 |
|  |  |  |  | (0.29-5.44) |  | (0.16-1.71) |  | (0.20-1.49) |  |
|  | *35-44* |  |  | *3.36*** | *0.064* | 0.97 | 0.958 | 1.28 | 0.569 |
|  |  |  |  | *(0.93-12.15)* |  | (0.36-2.66) |  | (0.55-2.98) |  |
|  | *45-54* |  |  | *3.12*** | *0.085* | 1.24 | 0.675 | 1.17 | 0.714 |
|  |  |  |  | *(0.865-11.42)* |  | (0.45-3.39) |  | (0.50-2.77) |  |
|  | 55+ |  |  | 2.67 | 0.221 | 0.37 | 0.269 | 0.97 | 0.964 |
|  |  |  |  | (0.55-12.91) |  | (0.06-2.16) |  | (0.30-3.15) |  |
| **Gender** | Male vs | *4.43*** | *0.052* | **3.41****** | **<0.001** | **4.15****** | **<0.001** | **3.68****** | **<0.001** |
|  | female | *(0.99-19.86)* |  | **(1.93-6.02)** |  | **(2.27-7.59)** |  | **(2.25-6.01)** |  |
| *SES quantile* | Per | *2.11** | *0.169* |  |  | *1.33** | *0.164* | *1.37*** | *0.059* |
|  | decreasing | *(0.73-6.14)* |  |  |  | *(0.89-1.97)* |  | *(0.99-1.89)* |  |
|  | quantile |  |  |  |  |  |  |  |  |
| **Years lived**  **in Am. Samoa** | <9 yrs |  |  | Ref |  | Ref |  | Ref |  |
|  | 9-14 yrs |  |  | **3.21***** | **0.033** | 1.60 | 0.330 | *2.26*** | *0.063* |
|  |  |  |  | **(1.10-9.42)** |  | (0.62-4.15) |  | *(0.96-5.34)* |  |
|  | >14 yrs |  |  | 1.80 | 0.215 | 0.73 | 0.433 | 1.27 | 0.502 |
|  |  |  |  | (0.71-4.56) |  | (0.34-1.59) |  | (0.63-2.57) |  |
| *Ever been* | Yes vs | *0.32** | *0.198* |  |  |  |  |  |  |
| *outside AS* | no | *(0.05-1.83)* |  |  |  |  |  |  |  |
| *Know* | Yes vs | *0.39** | *0.269* |  |  |  |  |  |  |
| *swelling or* | no | *(0.07-2.06)* |  |  |  |  |  |  |  |
| *genitourinary* |  |  |  |  |  |  |  |  |  |
| *symptoms* |  |  |  |  |  |  |  |  |  |
| **Know acute** | Yes vs | **5.15***** | **0.027** |  |  |  |  | *1.55*** | *0.167* |
| **symptoms** | no | **(1.21-21.95)** |  |  |  |  |  | *(0.83-2.87)* |  |
| **Know** | Yes vs |  |  | *0.59*** | *0.075* | **0.47***** | **0.012** | **0.47****** | **0.003** |
| **mosquito as** | no |  |  | *(0.34-1.05)* |  | **(0.26-0.85)** |  | **(0.29-0.77)** |  |
| **cause** |  |  |  |  |  |  |  |  |  |
| **Constant** |  | **0.005****** | **<0.001** | **0.03****** | **<0.001** | **0.15***** | **0.024** | **0.09****** | **0.001** |
|  |  | **(0.00008-0.22)** |  | **(0.005-0.18)** |  | **(0.03-0.77)** |  | **(0.02-0.38)** |  |
| log likelihood |  | -39.14823 |  | -175.21793 |  | -166.22426 |  | --222.59807 |  |
| log likelihood (inc. county) |  | -34.129366 |  | -171.04617 |  | -161.05818 |  | -218.04205 |  |
| LR Chi^2^ |  | **23.22***** | **0.039** | **42.89****** | **0.0003** | **53.91****** | **<0.001** | **58.92****** | **<0.001** |
| Pseudo R^2^ |  | 0.2538 |  | 0.1114 |  | 0.1434 |  | 0.1190 |  |

** p<0.2; ** p<0.1;* ***** p<0.05; **** p<0.01**
